# A Plant Virus Ensures Viral Stability in the Hemolymph of Vector Insects through Suppressing Prophenoloxidase Activation

**DOI:** 10.1128/mBio.01453-20

**Published:** 2020-08-18

**Authors:** Xiaofang Chen, Jinting Yu, Wei Wang, Hong Lu, Le Kang, Feng Cui

**Affiliations:** a State Key Laboratory of Integrated Management of Pest Insects and Rodents, Institute of Zoology, Chinese Academy of Sciences, Beijing, China; b CAS Center for Excellence in Biotic Interactions, University of Chinese Academy of Sciences, Beijing, China; EPFL

**Keywords:** plant virus, rice stripe virus, NS3, vector insect, small brown planthopper, prophenoloxidase, hemolymph

## Abstract

Most plant viruses require vector insects for transmission. Viral stability in the hemolymph of vector insects is a prerequisite for successful transmission of persistent plant viruses. However, knowledge of whether the proteolytic activation of prophenoloxidase (PPO) affects the stability of persistent plant viruses remains elusive. Here, we explored the interplay between rice stripe virus (RSV) and the PPO cascade of the vector small brown planthopper. Phenoloxidase (PO) activity was suppressed by RSV by approximately 60%. When the PPO cascade was activated, we found distinct melanization around RSV particles and serious damage to viral stability in the hemolymph. Viral suppression of PO activity was derived from obstruction of proteolytic cleavage of PPOs by binding of the viral nonstructural protein NS3. These results indicate that RSV attenuates the PPO response to ensure viral stability in the hemolymph of vector insects. Our research provides enlightening cues for controlling the transmission of vector-borne viruses.

## Abstract (precis)

Large ratios of vector-borne plant viruses circulate in the hemolymph of their vector insects before entering the salivary glands to be transmitted to plants. The stability of virions in the hemolymph is vital in this process. Activation of the proteolytic prophenoloxidase (PPO) to produce active phenoloxidase (PO) is one of the major innate immune pathways in insect hemolymph. How a plant virus copes with the PPO immune reaction in its vector insect remains unclear. Here, we report that the PPO affects the stability of rice stripe virus (RSV), a notorious rice virus, in the hemolymph of a vector insect, the small brown planthopper. RSV suppresses PPO activation using viral nonstructural protein. Once the level of PO activity is elevated, RSV is melanized and eliminated from the hemolymph. Our work gives valuable clues for developing novel strategies for controlling the transmission of vector-borne plant viruses.

## INTRODUCTION

Similarly to arboviruses infecting humans or animals, transmission of many plant viruses is largely fulfilled by vector insects. In 65 genera of vector-borne plant viruses, 54% of the viruses are of the persistent type, in which viruses enter the body of vectors and disseminate to various tissues (1). Many notorious plant viruses are persistent viruses, such as rice stripe virus, rice dwarf virus, and tomato yellow leaf curl virus. Persistently propagative plant viruses replicate within cells and circulate in the hemolymph of vector insects after successfully overcoming multiple tissue or membrane barriers (2). The stability of virions in the hemolymph is vital for the systemic dissemination of persistent viruses before entry into the salivary glands, from which they are transmitted to plants (3). Differing from the well-known cytoplasmic antiviral immune pathways that include small interfering RNAs, Imd, Toll, JAK-STAT, and autophagy (4, 5), one of major innate immune systems in insect hemolymph is the proteolytic activation of prophenoloxidase (PPO) (6, 7). Most studies on the PPO activation pathway reveal its function against the infection of pathogenic bacteria and fungi in invertebrates (8, 9). The relationship between virus fate and the PPO activation pathway in vector insects has not been sufficiently investigated.

In fact, the PPO activation pathway consists of a cascade of clip-domain serine proteases, which are activated after recognition of pathogens and which convert the zymogen PPO to active phenoloxidase (PO). PO catalyzes the conversion of monophenols to quinones, which form melanins restricting the activity of or killing pathogens (10). This pathway is negatively regulated by serpins, which maintain the PPO cascade in an inactive state when there is no immune challenge (11). Pathogenic bacteria and fungi usually induce the activation of PPO cascade, and some of them, such as Enterobacter cloacae or Micrococcus luteus, are frequently used as activators of this cascade in studies (12, 13).

Rice (Oryza sativa) is a requisite food for more than half of the world’s population. Rice stripe virus (RSV), a single-stranded RNA (ssRNA) virus of the genus *Tenuivirus*, causes one of the most destructive rice diseases and has resulted in severe yield losses in over 80% of the rice fields in eastern Asian countries (14, 15). RSV is specifically transmitted by the small brown planthopper Laodelphax striatellus in a persistent-propagative mode (16). The genome of RSV contains four RNA segments and encodes an RNA-dependent RNA polymerase (RdRp), a capsid protein (CP), and five nonstructural proteins (NS2, NSvc2, NS3, SP, and NSvc4) (17). Our previous study showed that several genes in the PPO activation pathway were differentially expressed in the salivary glands or gut of viruliferous insects (18), suggesting that the PPO activation pathway might be involved in the RSV transmission process.

In this study, we attempted to explore the mechanisms of molecular interactions between RSV and the PPO activation pathway of the small brown planthopper through investigating gene expression and PO activity variation. Interference with gene expression and bacterial stimulation were applied to further confirm the negative regulation of PO activity by RSV. Interactions between viral proteins and PPOs show that viral suppression of PO activity is derived from obstruction of proteolytic cleavage of PPOs. Such manipulation of the PPO activation pathway by RSV mediates viral stability in the hemolymph and is crucial for the RSV transmission process.

## RESULTS

### Transcriptional response of PPO activation pathway genes to RSV in two immune organs.

**FIG 1:**
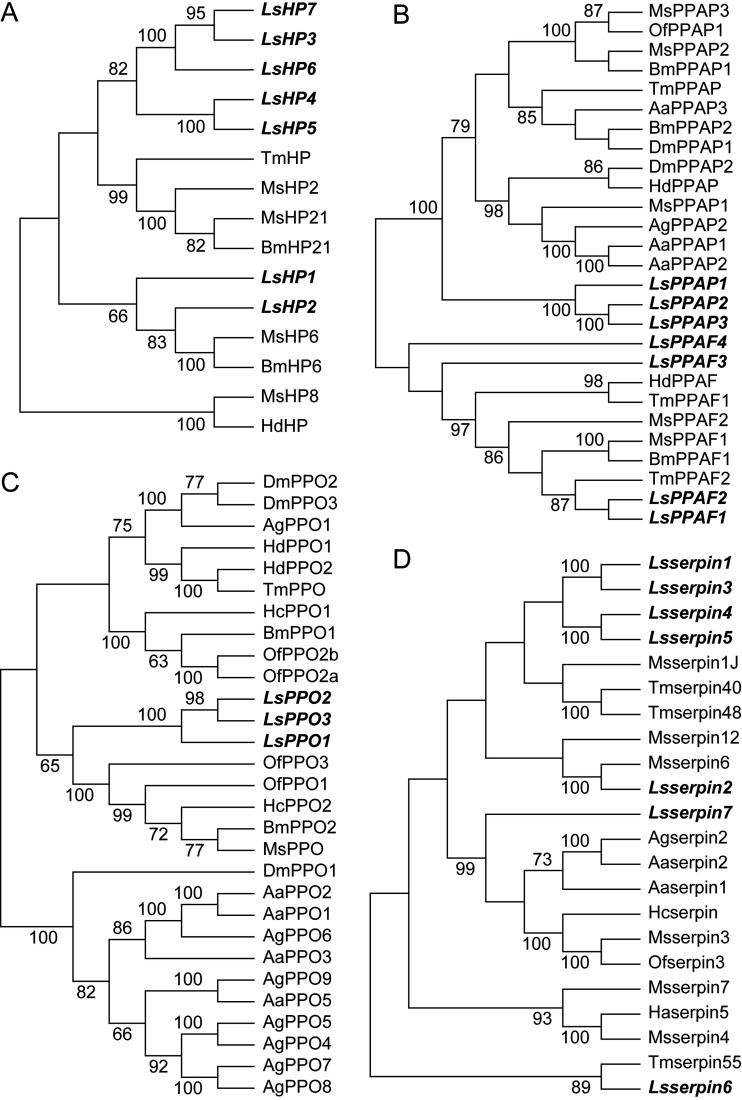
Phylogenetic trees of PPO activation cascade members of the small brown planthopper and other insect species. The neighbor-joining method (pairwise deletion and p-distance model) was used. Bootstrap analysis (1,000 replicates) was applied to evaluate the internal support of the tree topology. Bootstrap values higher than 60% were present at the nodes. (A) HPs. (B) PPAPs/PPAFs. (C) PPOs. (D) Serpins. The cascade members of the small brown planthopper are highlighted in italics and bold letters. The GenBank accession numbers of all of the proteins are listed in Table S1. Aa, Aedes aegypti; Ag, Anopheles gambiae; Bm, Bombyx mori; Dm, Drosophila melanogaster; Hd, Holotrichia diomphalia; Ha, Helicoverpa armigera; Hc, Hyphantria cunea; Ls, Laodelphax striatellus; Ms, Manduca sexta; Of, Ostrinia furnacalis; Tm, Tenebrio molitor.

The PPO activation pathway in insects contains hemolymph proteases (HPs), prophenoloxidase-activating proteases/factors (PPAPs/PPAFs), PPOs, and serpins (see Fig. S1 in the supplemental material). These components were searched for in the protein set of the small brown planthopper (19) through alignment with the characterized homologous proteins of 10 insect species (see Table S1 in the supplemental material) and further confirmed through phylogenetic analysis. Seven HPs (Fig. 1A), seven PPAPs/PPAFs (Fig. 1B), three PPOs (Fig. 1C), and seven serpins (Fig. 1D) were identified in the small brown planthopper.

10.1128/mBio.01453-20.1 FIG S1 Expression of PPO activation pathway genes not responsive to RSV in the fat body and hemocytes of planthoppers measured by quantitative real-time PCR. The relative transcript levels of the genes compared to that of *EF2* are reported as means ± SE. Fat bodies from 20 to 30 individuals and hemocytes from 100 to 150 individuals in a single replicate and in eight replicates for each organ were used. FB, fat body. H, hemocytes. No significant difference was observed for these genes between nonviruliferous and viruliferous insects. Download FIG S1, PDF file, 0.3 MB. Copyright © 2020 Chen et al. 2020 Chen et al. This content is distributed under the terms of the Creative Commons Attribution 4.0 International license.

10.1128/mBio.01453-20.5 TABLE S1 GenBank accession numbers of PPO activation cascade members of the small brown planthopper and other insect species. Download Table S1, DOCX file, 0.02 MB. Copyright © 2020 Chen et al. 2020 Chen et al. This content is distributed under the terms of the Creative Commons Attribution 4.0 International license.

**FIG 2:**
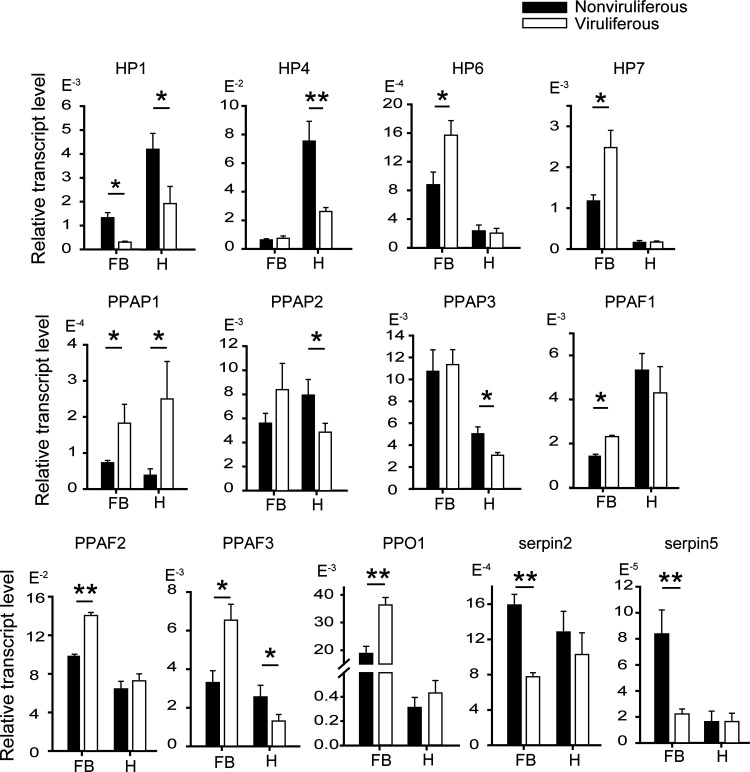
Differential responses of PPO activation pathway genes to RSV in the fat body and hemocytes of planthoppers measured by quantitative real-time PCR. The relative transcript level of each gene to that of *EF2* is reported as the mean ± SE. Fat bodies from 20 to 30 individuals and hemocytes from 100 to 150 individuals in a replicate and eight replicates for each organ were used. FB, fat body. H, hemocytes. *, *P* < 0.05; **, *P* < 0.01.

The gene response of the PPO activation pathway to RSV was checked in the fat body and hemocytes of viruliferous and nonviruliferous planthoppers using quantitative real-time PCR (qRT-PCR) (Fig. 2; see also Fig. S1). In the fat body, compared to those in the nonviruliferous planthoppers, the transcript levels of *HP6*, *HP7*, *PPAP1*, *PPAF1*, *PPAF2*, *PPAF3*, and *PPO1* were upregulated, and *HP1* and the transcript levels of inhibitor genes *serpin2* and *serpin5* were downregulated in the viruliferous planthoppers. In the hemocytes, the transcript levels of *HP1*, *HP4*, *PPAP2*, *PPAP3*, and *PPAF3* were downregulated, and only that of *PPAP1* was upregulated in the viruliferous planthoppers. RSV activated this pathway in fat bodies but inhibited it in hemocytes. Therefore, the transcriptional responses of the PPO activation pathway to RSV were different between the fat body and the hemocytes of vector insects.

### PO activity was inhibited by RSV in vector insects.

**FIG 3:**
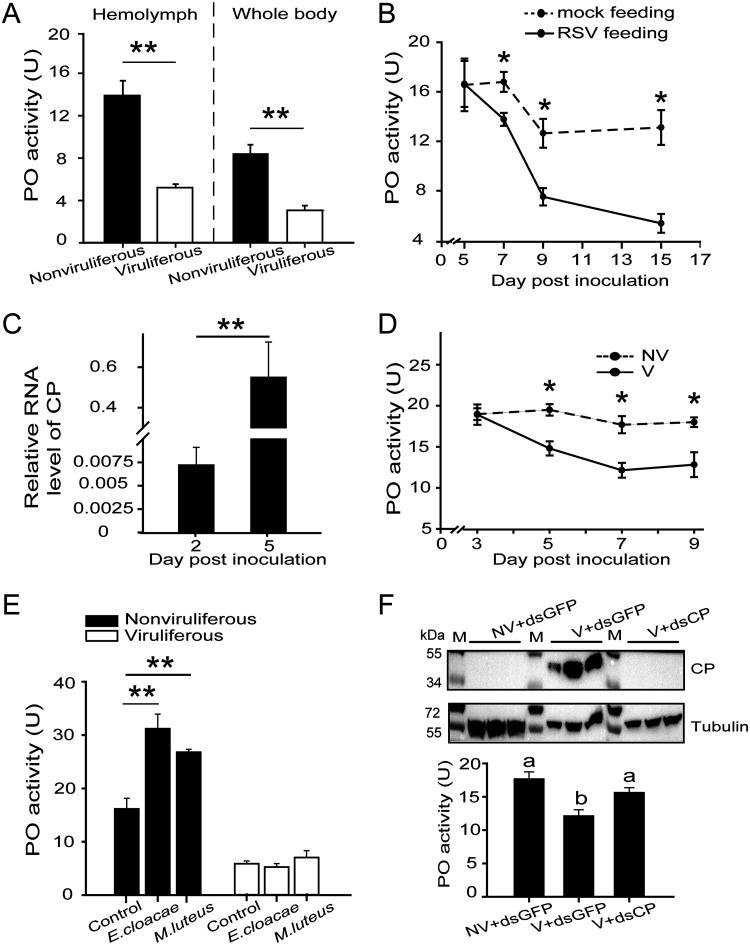
PO activity was inhibited by RSV in planthoppers. (A) PO activity in the hemolymph and whole body of viruliferous and nonviruliferous planthoppers. Totals of 10 and 12 replicates were used for hemolymph and whole-body samples, respectively. (B) PO activity was measured in the whole body of planthoppers fed on RSV-infected rice seedlings (RSV feeding), and the virus was allowed to incubate in insects for different times. The control group was nonviruliferous insects fed on rice seedlings without RSV infection (mock feeding). Six to nine replicates were used for each group. (C) Relative RNA levels of CP in the planthoppers injected with RSV crude preparations. Six to 10 replicates were tested for each group. (D) PO activity in the whole body of planthoppers injected with RSV crude preparations (V) or crude preparations from nonviruliferous insects (NV). Five to eight replicates were used for each group. (E) PO activity in nonviruliferous or viruliferous planthoppers infected with E. cloacae or M. luteus at 24 h. The control group was injected with water. Five or six replicates were used for each group. *, *P* < 0.05; **, *P* < 0.01. (F) Variations in CP and PO activity in planthoppers injected with the mixture of V and the double-stranded RNA of *CP* (ds*CP*). The control group was injected with a mixture of the double-stranded RNA of *GFP* (ds*GFP*) and V or NV. Five to eight replicates were used for each group. A homemade anti-CP polyclonal antibody was applied to quantify CP. An anti-human β-tubulin monoclonal antibody was used to measure tubulin as an internal control. Different letters indicate statistically significant differences in PO activity. M, marker.

Because zymogen PPOs are converted to active POs, we compared the PO activities of viruliferous and nonviruliferous planthoppers through surveying the production of melanin, using dopamine as a substrate. The levels of PO activities were 13.9 units (U) and 5.2 U in the hemolymph of nonviruliferous and viruliferous adult planthoppers and were 8.5 U and 3.1 U in the whole body, respectively (Fig. 3A). The enzymatic activity decreased by approximately 63% in viruliferous insects. Nonviruliferous insects were fed on RSV-infected rice seedlings for 3 days, and the virus was allowed to incubate in insects for 5 days, 7 days, 9 days, and 15 days postinoculation (dpi) from the beginning of the 3-day inoculation. The PO activity in the whole body of RSV-borne insects decreased from 7 dpi compared to that of nonviruliferous insects fed on healthy rice seedlings, and the maximum reduction was 59% at 15 dpi (Fig. 3B). When RSV crude preparations from viruliferous insects were microinjected into the hemolymph of nonviruliferous insects, viral replication was observed at 5 dpi (Fig. 3C), and the PO activity significantly decreased at 5, 7, and 9 dpi compared to that of the control group, which was injected with crude preparations from nonviruliferous insects (Fig. 3D). Thus, the PO activity of the planthoppers is reduced by RSV.

To further verify the influence of RSV on PO activity, we delivered E. cloacae (Gram-negative) or M. luteus (Gram-positive) bacteria into nonviruliferous and viruliferous adult planthoppers. The PO activity in nonviruliferous insects was induced in an obvious manner at 24 h after inoculation, while the enzymatic activity did not change in viruliferous insects subjected to the same bacterial treatments (Fig. 3E). Therefore, PO activity was still inhibited even though the planthoppers were coinfected with RSV and bacteria.

To counter the influence of RSV on PO activity, double-stranded RNA of CP (dsRNA-*CP*) was injected with RSV crude preparations into nonviruliferous insects. The viral amount was dramatically reduced at 7 dpi, as represented by detection of less CP than was measured after the injection of the mixture of ds*GFP*-RNA and the RSV crude preparation (Fig. 3F). The inhibition of viral replication resulted in increased PO activity, which attained the enzymatic level seen with nonviruliferous insects (Fig. 3F). All of the experiments mentioned above showed that RSV had an inhibitive effect on the PO activity of the vector insects.

### Adverse effects of POs on RSV stability in the hemolymph of vector insects.

**FIG 4:**
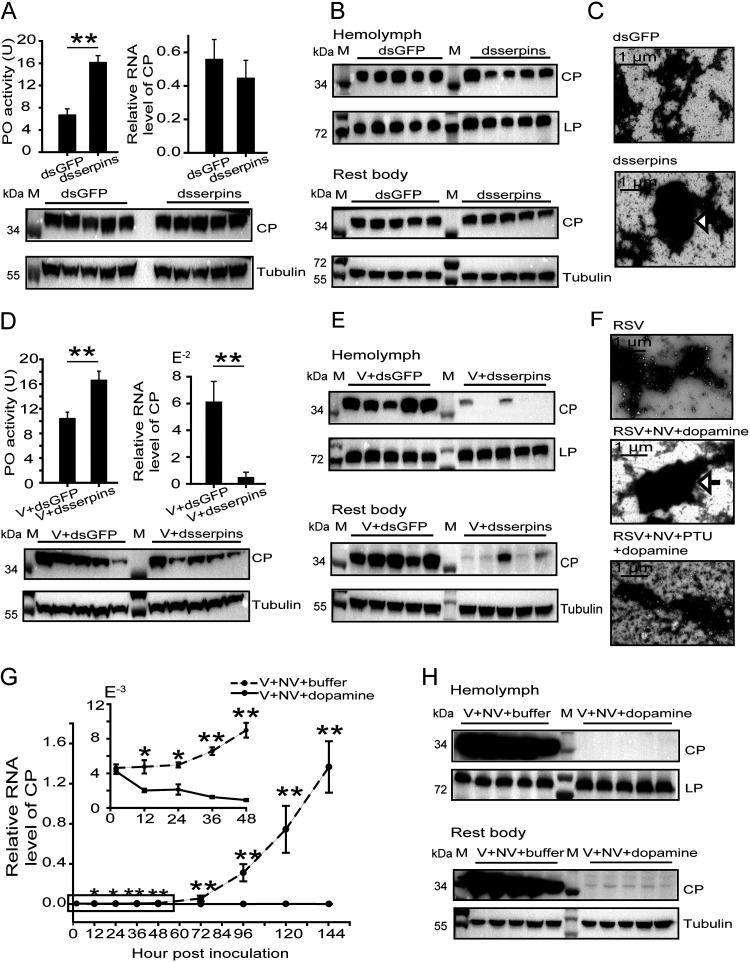
Adverse effects of POs on RSV stability in the hemolymph of planthoppers. (A) Variations in PO activity and CP RNA and protein levels in the whole body of viruliferous planthoppers after injection of the mixture of double-stranded RNAs of *serpin2* and *serpin7* (ds*serpins*) compared to those after injection of double-stranded RNA of *GFP* (ds*GFP*). The PO activity was measured with seven replicates. The RNA levels of *CP* were quantified with eight replicates. A homemade anti-CP polyclonal antibody was applied to quantify CP, and an anti-human β-tubulin monoclonal antibody was used to measure tubulin as an internal control via Western blotting. (B) Western blot to show CP in the hemolymph and rest body of viruliferous planthoppers after injection of ds*serpins* or ds*GFP*. A homemade anti-lipoprotein polyclonal antibody was used to quantify lipoprotein (LP) as an internal control. (C) Transmission electron microscopy images to show melanization around RSV particles isolated from the hemolymph of viruliferous planthoppers after injection of ds*serpins* or ds*GFP*. The arrow indicates melanized viruses. (D) Variations in PO activity and CP RNA and protein levels in the whole body of planthoppers 4 days after injection of a mixture of RSV crude preparations (V) and ds*serpins* or ds*GFP*. PO activity was assayed with eight replicates. The RNA levels of *CP* were quantified with six or seven replicates. (E) Western blot to show CP in the hemolymph and rest body of planthoppers injected with a mixture of V and dss*erpins* or ds*GFP*. (F) Transmission electron microscopy images to show melanization around purified RSV particles that were incubated with crude preparations from nonviruliferous planthoppers (NV) and dopamine in the presence or absence of phenylthiourea (PTU). The arrow indicates melanized viruses. (G) qRT-PCR showing the relative RNA levels of CP in planthoppers injected with a mixture of V, NV, and dopamine at different time points with 6 to 12 replicates. Dopamine was replaced by Tris-HCl buffer in the control group. The boxed time points are magnified in the upper-left panel. (H) Western blot to show CP in the hemolymph and rest body of planthoppers injected with a mixture of V, NV, and dopamine or Tris-HCl buffer at 144 h postinoculation. M, marker. *, *P* < 0.05; **, *P* < 0.01.

To investigate the effect of PO activity on virus fate in vector insects, two inhibitors of PPO activation, *serpin2* and *serpin7*, were manipulated. In viruliferous planthoppers, knocking down *serpin2* and *serpin7* using a mixture of ds*serpin2*-RNA and ds*serpin7*-RNA increased PO enzymatic activity (Fig. 4A). Although the elevated PO activity did not affect the viral load in the whole body at the CP RNA and protein levels (Fig. 4A), the viral load in the hemolymph significantly decreased at the CP level, but there was no difference in viral load in the rest body (Fig. 4B; see also Fig. S2A). After RSV particles were purified from the hemolymph using ultracentrifugation, distinct melanization around RSV particles was observed in the *serpin* knockdown group but not in the ds*GFP*-RNA injection group under a transmission electron microscope (Fig. 4C). When a RSV crude preparation was injected with the mixture of ds*serpin2*-RNA and ds*serpin7*-RNA into nonviruliferous planthoppers, PO activity was activated, and the viral load in the whole body, hemolymph, or the rest body was dramatically reduced at 4 dpi compared to the levels seen with the control groups (Fig. 4D and E; see also Fig. S2B).

**FIG 5:**
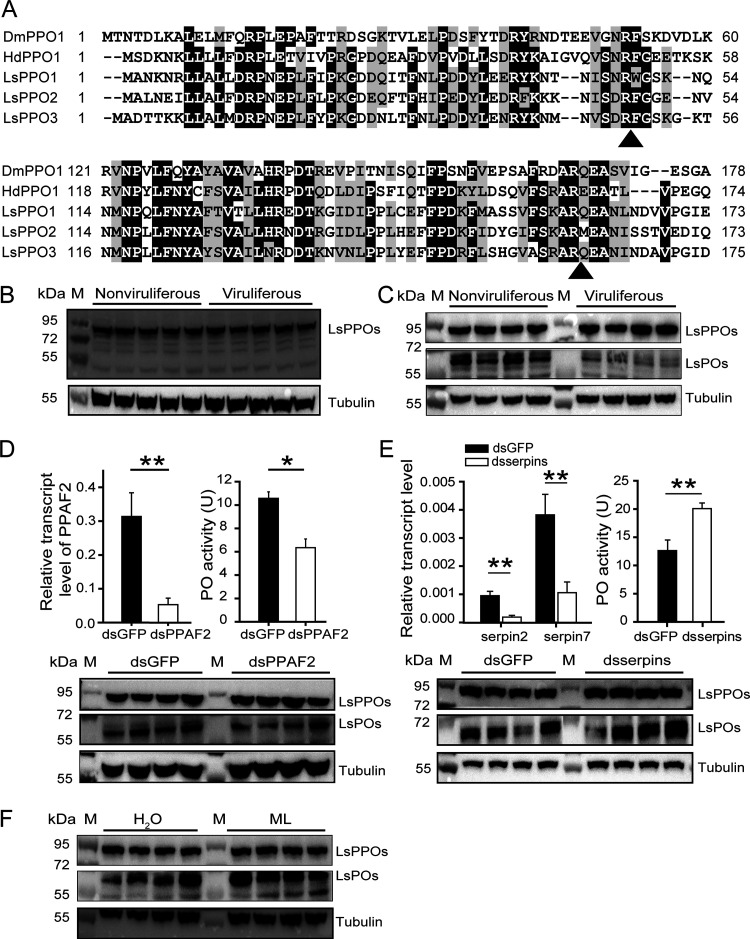
Reduction in PO production by RSV in planthoppers. (A) Sequence alignment of the PPO N terminus of several insects. Possible proteolytic cleavage sites are marked with arrows. Ls, L. striatellus. Dm, D. melanogaster. Hd, H. diomphalia. (B) Western blot assay showing the protein levels of LsPPOs and LsPOs in nonviruliferous and viruliferous planthoppers using an anti-LsPPO antibody. An anti-human β-tubulin monoclonal antibody was used to measure tubulin as an internal control. (C) Western blot assay showing the protein levels of LsPPOs and LsPOs by separate blottings. The PVDF membrane was excised at the site of the 72-kDa marker, and then the two pieces of membrane were separately incubated with the anti-LsPPO antibody. (D) Variations in PO activity and PO protein level in nonviruliferous planthoppers after injection of double-stranded RNAs of *PPAF2* (ds*PPAF2*) compared to those after injection of double-stranded RNA of *GFP* (ds*GFP*). PO activity was assayed with six replicates. The relative transcript level of *PPAF2* compared to that of *EF2* was measured by qRT-PCR with six replicates and reported as the mean ± SE. (E) Variations in PO activity and PO protein level in nonviruliferous planthoppers after injection of a mixture of double-stranded RNAs of *serpin2* and *serpin7* (dsserpins) compared to those after injection of ds*GFP*. PO activity was assayed with five replicates. The relative transcript level of *serpin2* and *serpin7* compared to that of *EF2* was measured by qRT-PCR with seven or eight replicates and reported as the mean ± SE. (F) Western blot assay showing the protein levels of LsPOs in nonviruliferous planthoppers that were inoculated with M. luteus (ML) or water. M, marker. *, *P* < 0.05; **, *P* < 0.01.

**FIG 6:**
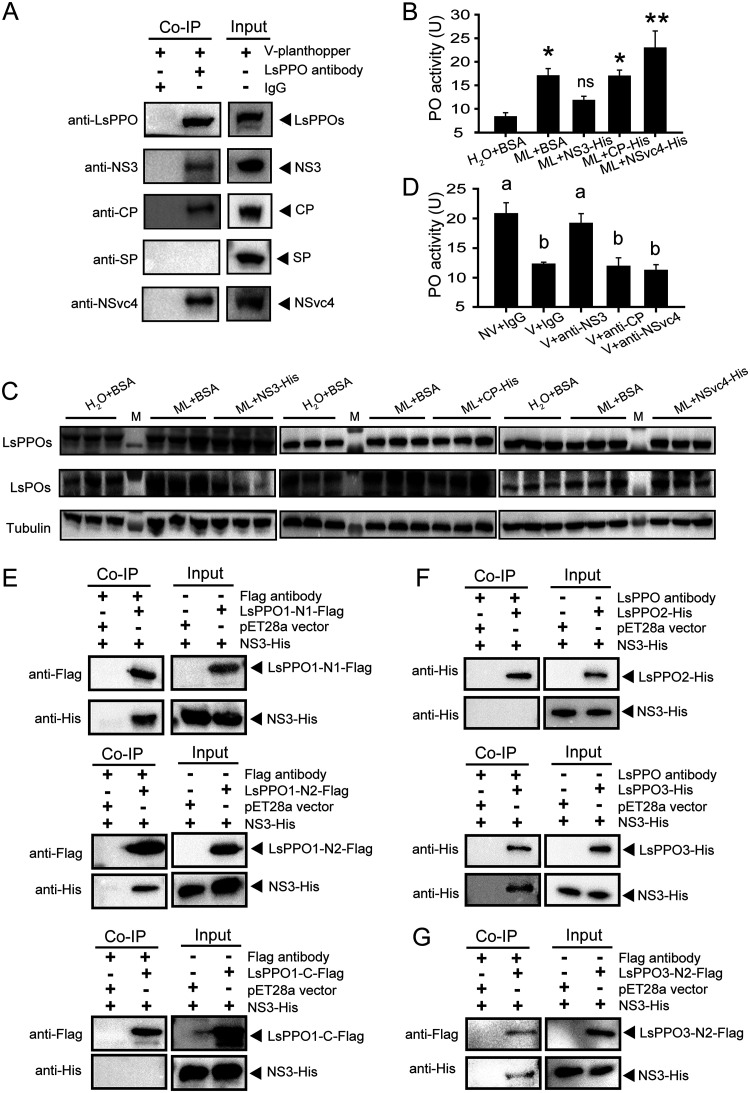
NS3 was responsible for the reduction in PO production. (A) Coimmunoprecipitation (Co-IP) assay for the interaction between LsPPOs and RSV proteins *in vivo* using an anti-LsPPO polyclonal antibody. Rabbit IgG was used as a negative control. Homemade monoclonal anti-CP, anti-NS3, and anti-SP antibodies and polyclonal anti-NSvc4 and anti-LsPPO antibodies were used in Western blot analysis. V-planthopper, the total proteins from viruliferous adult planthoppers. (B) Comparisons of PO activity in nonviruliferous planthoppers injected with a mixture of M. luteus (ML) and recombinantly expressed NS3-His, CP-His, NSvc4-His, or BSA compared to that of the control group insects injected with BSA and water. Seven to 10 replicates were used. *, *P* < 0.05; **, *P* < 0.01. ns, no significant difference. (C) Western blot assay showing the variation in LsPOs in the nonviruliferous planthoppers that were injected with a mixture of ML and NS3-His, CP-His, or NSvc4-His. The control group was injected with BSA and water or ML and BSA. An anti-LsPPO polyclonal antibody and anti-human β-tubulin monoclonal antibody were used. M, marker. (D) Comparisons of PO activity among planthoppers injected with a mixture of crude preparations from nonviruliferous planthoppers (NV) and IgG, RSV crude preparations (V) and IgG, V and anti-NS3 polyclonal antibody, V and anti-CP polyclonal antibody, or V and anti-NSvc4 polyclonal antibody. Five to seven replicates were used. Different letters indicate statistically significant differences in PO activity. (E) Co-IP assay for the interaction between a recombinantly expressed fragment of LsPPO1 (LsPPO1-N1-Flag, LsPPO1-N2-Flag, or LsPPO1-C-Flag) and NS3-His using an anti-Flag monoclonal antibody. The total proteins from E. coli expressing empty pET28a vector were used as a negative control. An anti-Flag or anti-His monoclonal antibody was used in Western blot analysis. (F) Co-IP assay for the interaction of recombinantly expressed LsPPO2-His or LsPPO3-His with NS3-His using an anti-LsPPO polyclonal antibody. (G) Co-IP assay for the interaction between LsPPO3-N2-Flag and NS3-His using an anti-Flag monoclonal antibody.

10.1128/mBio.01453-20.2 FIG S2 Densitometry analysis for CP or LsPO image bands. (A) Relative densities of CP in the experiments represented in Fig. 4B. (B) Relative densities of CP in the experiments represented in Fig. 4D. (C) Relative densities of LsPOs in the experiments represented in Fig. 5D. (D) Relative densities of LsPOs in the experiments represented in Fig. 5E. (E) Relative densities of LsPOs in the experiments represented in Fig. 5F. (F) Relative densities of LsPOs in the experiments represented in Fig. 5C. (G) Relative densities of LsPOs in the experiments represented in Fig. 6C. The density of CP or LsPOs was normalized to that of tubulin or lipoprotein, and data are presented as means ± SE. *, *P* < 0.05. Download FIG S2, PDF file, 0.3 MB. Copyright © 2020 Chen et al. 2020 Chen et al. This content is distributed under the terms of the Creative Commons Attribution 4.0 International license.

After the purified RSV particles were incubated with crude preparation from nonviruliferous planthoppers and the substrate dopamine for 3 h at 25°C, a great deal of melanin accumulated around RSV particles (Fig. 4F). This melanization was suppressed in the presence of phenylthiourea (PTU), a specific inhibitor of POs (Fig. 4F). When the melanin-trapped RSV particles were injected into nonviruliferous planthoppers, the CP RNA level in the whole body did not increase with time; instead, it showed a downward trend, in contrast to the remarkable rate of viral replication in the control group (Fig. 4G). Consequently, CP was only minimally detectable in the hemolymph and the rest body of the insects at 144 h postinjection with dopamine-treated RSV (Fig. 4H).

These data indicated that the activated PPOs were able to induce melanization, which was detrimental to the stability of RSV in the hemolymph and suppressed viral replication. Therefore, the ability to inhibit PO activity is crucial for RSV spread in vector insects.

### Reduction in PO production by RSV in vector insects.

Considering that PO activity is related to the amount of PO proteins, we measured the protein levels of PPOs and POs in nonviruliferous and viruliferous planthoppers. The three PPOs of L. striatellus (LsPPOs) had over 68% amino acid sequence identity, with a size of approximately 79 kDa. On the basis of alignment with Drosophila melanogaster and Holotrichia diomphalia PPOs, whose cleavage sites for proteolytic activation were determined previously (20, 21), the cleavage sites of LsPPOs were predicted. The first candidate cleavage sites were residue R48 for LsPPO1 and LsPPO2 and residue R49 for LsPPO3, potentially producing 74-kDa LsPOs (Fig. 5A). The second candidate cleavage site was R162 for the three LsPPOs, putatively producing 61-kDa LsPOs (Fig. 5A). An anti-LsPPO polyclonal antibody was generated using an *in vitro*-expressed fragment of LsPPO1-antigen (from amino acid residues 174 to 500) as the antigen, which showed over 70% identity to the corresponding fragments of LsPPO2 and LsPPO3. This antibody was able to recognize all three *in vitro*-expressed LsPPOs in the Western blot assay (Fig. S3).

10.1128/mBio.01453-20.3 FIG S3 Western blot assay performed to show the specificity of a homemade anti-LsPPO polyclonal antibody by recognition of *in vitro*-expressed LsPPO1-His, LsPPO2-His, and LsPPO3-His. E. coli transfected with pET28a vector was used as a negative control. M, marker. Download FIG S3, PDF file, 0.3 MB. Copyright © 2020 Chen et al. 2020 Chen et al. This content is distributed under the terms of the Creative Commons Attribution 4.0 International license.

When the anti-LsPPO antibody was applied to detect the protein levels of LsPPOs and LsPOs in the crude proteins from nonviruliferous and viruliferous planthoppers, a dark 79-kDa band corresponding to the size of LsPPOs appeared, and the levels of band intensity were similar between nonviruliferous and viruliferous planthoppers, suggesting similar protein levels of PPOs (Fig. 5B). However, LsPO proteins with a putative size of 74 kDa or 61 kDa were not clearly observed. To eliminate the effect of LsPPOs on LsPOs in the Western blot, we excised the polyvinylidene difluoride (PVDF) membrane at the site of the 72-kDa marker, and then the two pieces of membrane were separately incubated with the anti-LsPPO antibody. Subsequently, a band of 61 kDa appeared (Fig. 5C), indicating the presence of LsPO candidates.

Then, we conducted three experiments to verify LsPO candidates. As the expression of the PPO activation factor *PPAF2* was interfered with by the injection of ds*PPAF2*-RNA in nonviruliferous planthoppers, the quantity of 61-kDa LsPO candidates and the level of PO activity significantly decreased (Fig. 5D; see also Fig. S2C). On the other hand, when expression of *serpin2* and *serpin7* was knocked down using the mixture of ds*serpin2*-RNA and ds*serpin7*-RNA, the protein level of LsPO candidates and the PO activity dramatically increased (Fig. 5E; see also Fig. S2D). Furthermore, the inoculation of M. luteus in nonviruliferous insects remarkably promoted increases in the number of LsPO candidates after 24 h (Fig. 5F; see also Fig. S2E), consistent with the induced PO activity (Fig. 3E). Therefore, the band of 61-kDa proteins was confirmed as representing LsPOs and the cleavage site of LsPPOs was R162. The LsPO protein levels were much lower in viruliferous planthoppers than in nonviruliferous insects (Fig. 5C; see also Fig. S2F), confirming that RSV had inhibited PO production from zymogen PPOs.

### NS3 was responsible for the reduction in PO production.

To further reveal the molecular mechanisms underlying the inhibition of PO production from proteolyzed PPOs by RSV, the viral proteins binding to LsPPOs were pulled down from viruliferous planthoppers through a coimmunoprecipitation assay using the anti-LsPPO polyclonal antibody. Homemade monoclonal anti-CP, anti-NS3, and anti-SP antibodies and the polyclonal anti-NSvc4 antibody (22) were applied to detect the viral proteins in the pulldown products. The results showed that CP, NS3, and NSvc4 but not SP were precipitated with LsPPOs from the viruliferous planthoppers (Fig. 6A). The interactions between recombinantly expressed LsPPO1-Flag and CP-His, NS3-His, or NSvc4-His but not SP-His were further confirmed using an *in vitro* coimmunoprecipitation assay (Fig. S4A). The results showed that CP, NS3, or NSvc4 may participate in the regulation of PPO proteolysis. To verify this point, *in vitro*-expressed and purified NS3, CP, or NSvc4 was injected together with M. luteus into nonviruliferous planthoppers. Only NS3 suppressed the induction of PO activity (Fig. 6B) and the production of POs (Fig. 6C; see also Fig. S2G) stimulated by M. luteus, while CP and NSvc4 did not affect bacterial stimulation of PO production (Fig. 6B and C; see also Fig. S2G). After an anti-CP, anti-NS3, or anti-NSvc4 antibody was injected with RSV crude preparation into nonviruliferous planthoppers, only the anti-NS3 antibody rescued the PO activity, which was inhibited by RSV at 8 dpi, whereas the other two antibodies did not influence the PO activity (Fig. 6D). Thus, NS3 was the main player in the regulation of PPO proteolysis.

10.1128/mBio.01453-20.4 FIG S4 Coimmunoprecipitation (Co-IP) assay of the interaction between recombinantly expressed LsPPO1-Flag and RSV proteins with a His tag (A) and between fragments of LsPPO1 (LsPPO1-N1-Flag, LsPPO1-N2-Flag, and LsPPO1-C-Flag) and CP-His or NSvc4-His (B). E. coli transfected with pET28a vector was used as a negative control. An anti-Flag or anti-His monoclonal antibody was used in Western blot analysis. Download FIG S4, PDF file, 0.6 MB. Copyright © 2020 Chen et al. 2020 Chen et al. This content is distributed under the terms of the Creative Commons Attribution 4.0 International license.

As the proteolytic cleavage site for the LsPPOs was R162, three fragments of LsPPO1 with a Flag tag were recombinantly expressed in Escherichia coli, i.e., N1 from the N terminus to amino acid residue 210; N2 from amino acid residue 100 to residue 210, with a narrower cleavage region; and C from amino acid residue 211 to the C terminus. An *in vitro* coimmunoprecipitation assay showed that NS3 bound to the N1 and N2 fragments but did not bind the C fragment of LsPPO1 (Fig. 6E), indicating that NS3 is able to occupy the cleavage site of LsPPOs to prevent PO production. Interestingly, CP and NSvc4 bound to N1 and C but did not bind to N2 (Fig. S4B), supporting the idea that the two proteins did not affect PO production. We further tested the interactions between NS3 and other two LsPPOs. The *in vitro* coimmunoprecipitation assay showed that NS3 bound to LsPPO3 (Fig. 6F) and its N2 fragment (from amino acid residue 102 to residue 212 with a cleavage site; Fig. 6G), but not to LsPPO2 (Fig. 6F). Therefore, RSV suppressed PPO proteolysis mainly through binding the cleavage sites of PPO1 and PPO3 with the nonstructural protein NS3.

## DISCUSSION

One of the key stages for persistent plant viruses and animal arboviruses is circulation in the insect hemolymph to reach various organs after viruses are released from the insect gut. During the circulation process, these viruses have to deal with the immune response in insect hemolymph. The PPO activation pathway is one of the primary immune systems in insect hemolymph. Although this pathway is well known to play an important role in defense against pathogenic fungi, bacteria, and viruses (6, 7), the involvement of this pathway in the control of plant virus transmission by vector insects has not been investigated. In this study, we report that a persistent plant virus utilizes its nonstructural protein to suppress PO production and melanization for stable viral circulation in vector insects.

In what was to our knowledge the first case study of the interplay between a plant virus and the PPO activation pathway of a vector insect, we found that RSV reduced PO activity by approximately 60% in viruliferous planthoppers. In contrast, pathogenic viruses also inhibit the PO activity of the host but to a larger degree. For example, the PO activity of white spot syndrome virus (WSSV)-infected shrimp decreased approximately 6-fold at 2 and 3 dpi compared to that of uninfected shrimp (23). The PO activity of Helicoverpa armigera was inhibited by baculovirus by 7.4-fold at 48 h postinoculation (hpi) and 4.2-fold at 72 hpi compared to the results seen with controls (24). Therefore, inhibition of insect humoral melanization seems to be a common characteristic for plant viruses and pathogenic viruses but the degrees of inhibition differ. Plant viruses show weaker suppression regulation of the insect PPO system than pathogenic viruses. This distinction could represent one of the important conditions accounting for the different replication levels of the two types of viruses in insects, i.e., limited replication of plant viruses in vector insects versus uncontrolled replication of pathogenic viruses in host insects.

The weak suppression of the insect PPO system has biological significance for plant viruses. When PPOs of planthoppers were artificially activated *in vivo* or *in vitro*, obvious melanization appeared with the ultracentrifugation-purified RSV particles trapped by melanin, and melanization resulted in instability of RSV in the hemolymph. However, this melanization phenomenon was not observed directly in the PPO-activated hemolymph because RSV particles were undetectable *in vivo*, probably due to the low viral load and inconsistencies in the shapes of the viruses. The melanization phenomenon was also not found in vector insects carrying other persistent plant viruses or arboviruses, perhaps because of the suppressive effect of the viruses on the PPO system. Although the extent of plant virus inhibition of the insect PPO system is not as large as that shown by pathogenic viruses, this weak suppression is enough to eliminate the possibility of melanization and to ensure stable circulation of plant viruses in insect hemolymph. In contrast, fungi and bacteria usually activate the PPO system and induce melanization, as shown by such species as Beauveria bassiana in Anopheles gambiae and Ostrinia furnacalis (9, 25), M. luteus and Saccharomyces cerevisiae in Bombyx mori (26), M. luteus and B. bassiana in *Drosophila* (27), and Staphylococcus aureus and Vibrio parahaemolyticus in Procambarus clarkii (28). Thus, viruses have effects opposite of those of fungi and bacteria on the PPO activation pathway of insects or other arthropods.

The nonstructural NS3 protein of RSV occupied the cleavage sites of PPOs, impeding PO production from PPOs. This “PPO suppressor” activity is a newly revealed function for NS3, in addition to its being a suppressor of RNA silencing in host plants and of the 26S proteasome-mediated defense response in vector insects (29, 30). In addition, CP and NSvc4 also bound PPOs, but they neither affected PPO cleavage nor inhibited PO activity. Their roles in the PPO system need further exploration. The regulation strategies of pathogenic viruses in hosts are different from those of RSV in vector insects. WSSV makes many efforts to inhibit the PPO system of shrimp: the viral protein WSSV453 binds proPPAP2 to interfere with its conversion to active PPAP2; the viral protein WSSV164 directly inhibits PO catalytic ability; and WSSV induces the expression of shrimp miR-315 to reduce the expression of PPAP3 (31–33). In H. armigera, baculovirus downregulated protein levels of most PPO cascade members but upregulated *serpin5* and *serpin9* expression in the hemolymph (24). Microplitis demolitor bracovirus is a symbiotic polydnavirus of the braconid wasp Microplitis demolitor. Two viral proteins, Egf1.0 and Egf1.5, inhibited PPAP release from proPPAP and its PPO digestion activity in Manduca sexta, which is the host of braconid wasps (34, 35). Therefore, different viruses have evolved different mechanisms to suppress the insect PPO immune pathway. Only by clarifying the exact mechanism for each specific virus can we exploit it for regulating the relation between viruses and hosts in an intended direction.

The members of the L. striatellus PPO activation cascade were identified based on sequence homology with those of other insects. HPs, PPAFs, PPOs, and serpins are comparatively conserved among insects, while three PPAPs of L. striatellus show high levels of divergence from orthologs of other insects, represented by only 29% to 37% identity in amino acid sequences with orthologs of other insects. Whether the three PPAPs possess PPO digestion activity requires experimental verification. The responses of most PPO cascade members to RSV infection were different in the two immune organs, as shown by activation of this pathway in the fat body and inhibition in hemocytes. Similar situations were also observed in other studies. For example, the transcript level of a *PPAP* of white shrimp, Litopenaeus vannamei, was suppressed in hemocytes and enhanced in gill in response to infection by Vibrio harveyi (36). *HP2* was downregulated in hemocytes but upregulated in the fat body of M. sexta after infection with E. coli (37). Why the PPO cascade behaves differently in the two immune organs in the presence of pathogens deserves further investigation.

In summary, we found that a persistent plant virus suppressed the function of PPO immune effectors in the hemolymph of vector insects to ensure virus stability during circulation in the hemolymph. This stage is vital for successful transmission of persistent plant viruses from vector insects to host plants. Stimulation of the insect PPO activation pathway would be a promising avenue for controlling the transmission of vector-borne viruses, such as through upregulating *HP*s, *PPAP*s, or *PPAF*s; inhibiting *serpin*s; or designing competitive inhibitors to block the binding of NS3 with PPOs.

## MATERIALS AND METHODS

### Small brown planthopper strains.

The viruliferous and nonviruliferous small brown planthopper strains used in this study were established from field populations collected from Jiangsu Province, China. The insects were reared on seedlings of Oryza sativa L. subsp. *japonica* var. *nipponbare* rice in glass incubators at different insectaries (38). To ensure that the RSV-carrying frequency was no less than 90%, the viruliferous strain was screened every 3 months via dot enzyme-linked immunosorbent assay (Dot-ELISA) with a monoclonal anti-CP antibody as described previously (38).

### Identification of PPO cascade members of L. striatellus.

Protein sequences of the PPO activation pathway proteins from Aedes aegypti, A. gambiae, B. mori, D. melanogaster, M. sexta, Tenebrio molitor, H. diomphalia, O. furnacalis, H. armigera, and Hyphantria cunea were applied for BLAST analysis in the gene set and transcriptome of L. striatellus (18, 19) using BLASTp with a cutoff E value of <10^−5^. The phylogenetic relationships of these L. striatellus candidate PPO cascade members to those from other insect species were analyzed with the neighbor-joining method (pairwise deletion and p-distance model) using Mega 6.06 software (RRID: SCR_000667). Bootstrap analysis (1,000 replicates) was applied to evaluate the internal support of the tree topology.

### Collection of fat bodies and hemolymph.

Adult planthoppers were gently laniated from abdomens with surgical forceps in 10 mM Tris-HCl buffer (pH 8.0) on a glass slide. The white fat bodies floating in the buffer were collected using a 0.5-to-10-μl pipette and transferred to 1.5-ml centrifuge tubes containing 100 μl of TRIzol reagent. Eight replicates and fat bodies from 20 to 30 individuals in a replicate were prepared.

To collect hemolymph, 30 adult planthoppers were gently laniated from abdomens with surgical forceps in 100 μl of 10 mM Tris-HCl buffer (pH 8.0) on a glass slide. The buffer and laniated planthoppers were transferred to a 500-μl centrifuge tube with a small hole at the bottom of the tube. The 500-μl centrifuge tube was put in a 1.5-ml centrifuge tube and centrifuged at 2,000 × *g* for 15 min at 4°C. The supernatant in the 1.5-ml outer tube was kept as clear hemolymph solution and used for PO activity assay. Ten replicates were prepared.

We removed legs of adult planthoppers with surgical scissors and gently pressed abdomens. Small drops of transparent hemolymph, containing hemocytes, exuded from wound sites and were collected into 1.5-ml centrifuge tubes with 100 μl of TRIzol reagent for total RNA isolation from hemocytes. Eight replicates and hemocytes from 100 to 150 individuals in a replicate were prepared.

### PO activity assay.

Twenty adult planthoppers were homogenized in 100 μl of 10 mM Tris-HCl buffer (pH 8.0) and centrifuged at 12,000 × *g* for 15 min at 4°C, and 65 μl of the supernatant was gently mixed with 100 μl of 4 mg/ml dopamine in 10 mM Tris-HCl buffer (pH 8.0) in a 96-well plate at 27°C for 10 min. Twelve replicates for viruliferous and nonviruliferous planthoppers were prepared. For analysis of PO activity from hemolymph, 65 μl of the clear hemolymph solution was mixed with 100 μl of 4 mg/ml dopamine. The absorbance of melanin was monitored at 490 nm (*A*_490_) by the use of a SpectraMax Paradigm reader (Molecular Devices, San Jose, CA, USA) every 5 min. The protein concentration of the supernatant was determined using the Bradford method. One unit (U) of PO activity was defined as 0.001 Δ*A*_490_ for every milligram protein in 1 min (39). Values representing the PO activity of each group are represented as means ± standard errors (SE). Differences were statistically evaluated in SPSS 17.0 using Student’s *t* test to compare two means or using one-way analysis of variance (ANOVA) followed by Tukey’s test for multiple comparisons.

### RNA isolation and cDNA synthesis.

Total RNA was isolated from fat bodies, hemocytes, or whole bodies (five as a replicate) using TRIzol reagent (Invitrogen, Carlsbad, CA, USA) according to the manufacturer’s protocol. RNA was treated with a Turbo DNA-free kit (Ambion, Austin, TX, USA) to eliminate genomic DNA contamination. One microgram of RNA was subjected to reverse transcription to cDNA using a Moloney murine leukemia virus (M-MLV) reverse transcription system (Promega, Madison, WI, USA) and random primers (Promega) following the manufacturer’s instructions.

### Quantitative real-time PCR.

Quantitative real-time PCR (qRT-PCR) was used to quantify the relative RNA levels of RSV *CP* and PPO cascade members of L. striatellus in the whole body or two immune organs. The primers for each gene are listed in Table S2 in the supplemental material. qRT-PCR was performed in 20 μl of reaction agent composed of 10 μl of 2× SYBR green PCR master mix (Fermentas, Waltham, MA, USA), 1 μl of cDNA template, and a 0.25 μM concentration of each primer by the use of a LightCycler 480 II system (Roche, Basel, Switzerland). The thermal cycling conditions were 95°C for 2 min followed by 40 cycles of 95°C for 30 s, 60°C for 30 s, and 68°C for 40 s. The transcript level of translation elongation factor 2 (*EF2*) was quantified as an internal reference to normalize the cDNA templates. The relative transcript level of each gene is reported as the mean ± SE. Differences were statistically evaluated using Student’s *t* test in SPSS 17.0.

10.1128/mBio.01453-20.6 TABLE S2 Primers used in this study. Download Table S2, DOCX file, 0.02 MB. Copyright © 2020 Chen et al. 2020 Chen et al. This content is distributed under the terms of the Creative Commons Attribution 4.0 International license.

### Double-stranded RNA synthesis and delivery.

Double-stranded RNAs (dsRNAs) for *PPAF2*, *serpin2*, *serpin7*, *CP*, and the green fluorescent protein gene (*GFP*) were synthesized using a T7 RiboMAX Express RNA interference (RNAi) system (Promega) following the manufacturer’s protocol and assessed by agarose gel electrophoresis for their purity and integrity. The corresponding PCR primers of dsRNA for these genes are listed in Table S2. Injection of 23 nl of dsRNAs at 6 μg/μl for each gene was performed on the adult planthoppers. The dsRNAs were delivered into the hemolymph in the ventral thorax by microinjection through a glass needle using a Nanoliter 2000 injector (World Precision Instruments, Sarasota, FL, USA).

### Interference with *serpin* and *PPAF2* expression.

Viruliferous adult planthoppers were injected with 23 nl of a mixture containing ds*serpin2*-RNA and ds*serpin7*-RNA at 6 μg/μl. ds*GFP*-RNA at 6 μg/μl was injected as a control. The transcript levels of the two *serpins* were quantified by qRT-PCR to calculate the interference rates at 4 dpi, with six replicates. The PO activity was measured at 4 dpi, with seven replicates. The RNA levels of *CP* in the whole body were quantified at 8 dpi, with eight replicates. The protein levels of CP in the whole body, hemolymph, and rest body were assayed at 8 dpi using Western blotting, with five replicates. The melanization of RSV particles isolated from the hemolymph was observed with a transmission electron microscope.

Nonviruliferous adult planthoppers were injected with 23 nl of ds*PPAF2*-RNA or a mixture containing ds*serpin2*-RNA and ds*serpin7*-RNA at 6 μg/μl. The control group was injected with 23 nl of ds*GFP*-RNA at 6 μg/μl. The insects were collected at 4 dpi. The interference rates of the three genes were quantified by qRT-PCR, with six to eight replicates. The PO activity was measured, with five or six replicates. The protein levels of LsPPO/LsPO were checked using Western blot analysis, with four replicates.

### Feeding small brown planthoppers with rice seedlings carrying RSV.

Nonviruliferous five-instar nymphs were fed on RSV-infected rice seedlings for 3 days and then transferred to healthy rice seedlings. The planthoppers were collected at 5, 7, 9, and 15 dpi for a PO activity assay. Another group of nonviruliferous nymphs were raised on healthy rice seedlings as negative controls. Six to nine replicates were prepared for each group.

### Injection of small brown planthoppers with RSV crude preparations.

Fifty viruliferous adult planthoppers were homogenized with a disposable polypropylene pestle in 100 μl of 10 mM Tris-HCl buffer (pH 8.0) contained in a 1.5-ml tube. After centrifugation at 12,000 × *g* for 15 min at 4°C, the supernatant was kept. The centrifugation was repeated four times in total, and the supernatant from the last centrifugation was used as the RSV crude preparation. A total of 23 nL of RSV crude preparations was injected into the hemolymph of nonviruliferous adult planthoppers through a glass needle using a Nanoliter 2000 injector. Injection of 23 nl of crude extracts from nonviruliferous adult planthoppers was used as a negative control. The planthoppers were collected at 3, 5, 7, and 9 dpi for the PO activity assay, with five to eight replicates for each group. The RNA level of CP in planthoppers was quantified with qRT-PCR at 2 and 5 dpi, with six to 10 replicates.

### Injection of small brown planthoppers with a mixture of RSV crude preparations and dsRNAs.

Equal aliquots of RSV crude preparations and ds*CP*-RNAs were mixed, and 23 nl of the mixture was injected into nonviruliferous adult planthoppers. Injections of a mixture of RSV crude preparations and ds*GFP*-RNA or a mixture of crude extracts from nonviruliferous adult planthoppers and ds*GFP*-RNA were used as negative controls. At 7 dpi, proteins were extracted from five planthoppers for Western blot analysis of CP amount, with three biological repeats. PO activity was also assayed with five to eight replicates.

Equal aliquots of RSV crude preparations, ds*serpin2*-RNAs, and ds*serpin7*-RNAs were mixed, and 23 nl of the mixture was injected into nonviruliferous adult planthoppers. Injection of a mixture of RSV crude preparations and ds*GFP*-RNA was used as a negative control. Total RNA was isolated from five planthoppers at 4 dpi to quantify the RNA level of *CP* using qRT-PCR, with six or seven replicates. Proteins were extracted at 4 dpi from whole bodies, hemolymph, and rest body for Western blot analysis of CP amount. PO activity in whole bodies was assayed with eight replicates.

### Injection of small brown planthoppers with a mixture of RSV crude preparations and antibodies.

Equal aliquots of RSV crude preparations and each polyclonal anti-CP, anti-NS3, or anti-NSvc4 antibody were mixed, and 23 nl of the mixture was injected into nonviruliferous adult planthoppers. At 4 dpi, the insects were injected with 32.2 nl of the corresponding polyclonal antibody a second time. The control groups were injected with a mixture of RSV crude preparations and IgG, with a mixture of crude preparations from nonviruliferous insects and IgG, or with IgG alone a second time. The planthoppers were collected at 7 dpi for the PO activity assay, with five to seven replicates.

### Injection of small brown planthoppers with bacterial and viral proteins.

E. cloacae was cultured to an optical density at 600 nm (OD_600_) of 0.15 and then diluted 1,000 times with water before use. Freeze-dried M. luteus was dissolved in water at 1 mg/ml. A total of 23 nl of E. cloacae or M. luteus was injected into nonviruliferous and viruliferous adult planthoppers. A control group was injected with water. PO activity was assayed at 24 hpi with five or six replicates. Proteins were extracted from five M. luteus-injected insects and from the control group at 24 hpi for LsPPO/LsPO detection using Western blot analysis. Four replicates were prepared.

In another experiment, M. luteus was mixed with *in vitro*-expressed and purified viral protein (NS3, CP, or NSvc4). Nonviruliferous adult planthoppers were injected with 23 nl of a mixture containing 4 mg/ml M. luteus and 2 mg/ml NS3, 46 nl of a mixture containing 2 mg/ml M. luteus and 0.25 mg/ml CP, or 46 nl of a mixture containing 2 mg/ml M. luteus and 1 mg/ml NSvc4. The control groups were injected with 4 mg/ml M. luteus and 2 mg/ml bovine serum albumin (BSA) or with water and 2 mg/ml BSA. Planthoppers were collected at 16 hpi for a PO activity assay, with 7 to 10 replicates, and for LsPPO/LsPO detection using Western blot analysis, with three replicates.

### Observation of RSV melanization and injection of melanized RSV.

Hemolymph was isolated from 100 viruliferous adult planthoppers injected with ds*serpins*-RNA or ds*GFP*-RNA in 1.5 ml of 10 mM Tris-HCl buffer (pH 8.0). After centrifugation at 10,000 × *g* and 4°C for 30 min, the supernatant was kept and ultracentrifuged at 100,000 × *g* and 4°C for 1 h. The pellet was suspended in 20 μl of 10 mM Tris-HCl buffer (pH 8.0). RSV crude preparations from the whole body were first centrifuged at 10,000 × *g* and 4°C for 30 min, and then the supernatant was ultracentrifuged at 100,000 × *g* and 4°C for 2 h. The pellet was suspended in 1.5 ml of 10 mM Tris-HCl buffer (pH 8.0) for a second ultracentrifugation for 2 h. The pellet was suspended in 30 μl of Tris-HCl buffer and incubated with 30 μl of nonviruliferous crude preparations and 30 μl of 4 mg/ml dopamine for 3 h at 25°C. In another group, 1 μl of a solution consisting of water and PTU (a specific inhibitor of PO) was added. Solutions were deposited on Formvar-carbon-coated electron microscopy grids, stained with 2% (wt/vol) uranyl acetate, and observed under an electron microscope (Tecnai G2 F20 Twin TMP; FEI, Eindhoven, Holland) at 80 kV.

Thirty microliters of RSV crude preparation from the whole body was incubated with 30 μl of nonviruliferous crude preparation and 30 μl of 4 mg/ml dopamine for 3 h at 25°C. Dopamine was replaced by Tris-HCl buffer in the control group. Then, 23 nl of the mixture was injected into nonviruliferous adult planthoppers. Insects were collected at 2, 12, 24, 36, 48, 72, 96, 120, and 144 hpi to quantify the RNA level of CP using qRT-PCR. Six to 12 replicates were prepared. Proteins were extracted from the hemolymph and rest body of planthoppers at 144 hpi to check the protein level of CP using Western blot assay.

### Western blot analysis for CP, LsPPOs, and LsPOs.

Proteins were extracted from five whole bodies, hemolymph, and the rest body of 20 viruliferous planthoppers or RSV-injected planthoppers in 50, 50, and 200 μl of 10 mM Tris-HCl buffer (pH 8.0), respectively, for Western blot analysis. A homemade anti-CP polyclonal antibody was applied to quantify CP. An anti-human β-tubulin monoclonal antibody (EASYBIO, Beijing, China) was used to measure tubulin as an internal control in whole-body and rest body samples. A homemade anti-lipoprotein polyclonal antibody was used to quantify lipoprotein as an internal control in hemolymph samples (40). The protein levels of LsPPOs and LsPOs in planthoppers were revealed using a homemade anti-LsPPO polyclonal antibody. To eliminate the effect of LsPPOs on LsPOs in the Western blot, the PVDF membrane was excised at the site of the 72-kDa marker, and then the two pieces of membrane were separately incubated with the anti-LsPPO antibody. The density of CP or LsPOs was quantified with Gelpro32 image analysis software and normalized to that of tubulin or lipoprotein. Differences were statistically evaluated in SPSS 17.0 using Student’s *t* test to compare two means or one-way ANOVA followed by Tukey’s test for multiple comparisons.

### Protein expression and purification and antibody preparation.

The full-length open reading frames (ORFs) of NS3, CP, SP, NSvc4, LsPPO1, LsPPO2, and LsPPO3 and a fragment of lipoprotein (amino acid residues 39 to 600, named lipoprotein-antigen) were amplified from a viruliferous planthopper cDNA library and ligated with PEGM-Teasy plasmid. A Flag tag was added to the 3′ terminus of LsPPO1, LsPPO1-N1 (amino acid residues 1 to 210), LsPPO1-N2 (100 to 210), LsPPO1-C (211 to 692), and LsPPO3-N2 (102 to 212) through PCR. A His tag from the pET28a vector was added to the 5′ terminus of NS3, CP, SP, NSvc4, LsPPO1, LsPPO1-antigen (residues 174 to 500), LsPPO2, LsPPO3, and lipoprotein-antigen (residues 39 to 600). The primer sequences for each gene are listed in Table S2. His-tagged fragments were inserted into the pET28a vector between restriction sites BamHI and XhoI and Flag-tagged fragments between NcoI and EcoRI through homologous recombination using an In-Fusion HD cloning kit (TaKaRa Bio USA, Inc., Mountain View, CA, USA). The recombinant pET28a plasmids were transformed into E. coli strain BL21(DE3) for protein expression. After overnight induction with 0.5 mM isopropyl β-d-thiogalactoside at 16°C, cells were collected by centrifugation and sonicated for 30 min in ice water. The supernatant was kept for coimmunoprecipitation assay and protein purification. The expressed recombinant proteins LsPPO1-antigen and lipoprotein-antigen were purified using Ni Sepharose (GE Healthcare, Buckinghamshire, United Kingdom) following the manufacturer’s instructions and served as antigens to produce anti-LsPPO and anti-lipoprotein rabbit polyclonal antibodies at Beijing Protein Institute Co., Ltd. (Beijing, China). The specificity of the anti-LsPPO antibody was tested by recognizing *in vitro*-expressed LsPPO1-His, LsPPO2-His, and LsPPO3-His in a Western blot assay.

### Coimmunoprecipitation assay.

Ten micrograms of rabbit anti-LsPPO polyclonal antibody was first incubated with 50 μl of Dynabeads protein G (Novex, Thermo Fisher Scientific, Waltham, MA, USA) for 10 min at room temperature, and then 400 μl of the total proteins from viruliferous adult planthoppers in 10 mM Tris-HCl buffer (pH 8.0) was added and incubated for 2 h at 4°C. Approximately 10% of the total protein was reserved as input. Rabbit IgG (Merck Millipore, Billerica, MA, USA) was used as a negative control. After three washes with washing buffer (Novex), the antibody-protein complex was disassociated from the beads with elution buffer (Novex) for Western blot analysis using homemade monoclonal anti-CP, anti-NS3, and anti-SP antibodies and polyclonal anti-NSvc4 and anti-LsPPO antibodies (22).

Five micrograms of mouse anti-Flag monoclonal antibody was incubated with 50 μl of Dynabeads protein G (Novex) for 10 min at room temperature. Then, 400 μl of a 1:1 mixture of recombinantly expressed LsPPO1-Flag and viral protein (NS3-His, CP-His, SP-His, or NSvc4-His) or a 1:1 mixture of a recombinantly expressed fragment of LsPPO1 (LsPPO1-N1-Flag, LsPPO1-N2-Flag, or LsPPO1-C-Flag) and viral protein (NS3-His, CP-His, or NSvc4-His) or a 1:1 mixture of recombinantly expressed LsPPO3-N2-Flag and NS3-His was added and the reaction mixture was incubated for 30 min at 4°C. Ten micrograms of rabbit anti-LsPPO polyclonal antibody was incubated with 50 μl of Dynabeads protein G (Novex) for 10 min at room temperature. Then, 400 μl of a 1:1 mixture of recombinantly expressed LsPPO2-His and NS3-His or LsPPO3-His and NS3-His was added and the reaction mixture was incubated for 30 min at 4°C. The total proteins from E. coli expressing empty pET28a were applied in the control group. After three washes performed with washing buffer (Novex), the antibody-protein complex was disassociated from the beads with elution buffer (Novex) for Western blot analysis performed with anti-Flag or anti-His monoclonal antibodies (CWBiotech, Beijing, China).

### Data availability.

GenBank accession numbers of PPO activation cascade members are listed in Table S1.
