# Supplementary figures and images for: A Plant Virus Ensures Viral Stability in the Hemolymph of Vector Insects through Suppressing Prophenoloxidase Activation
Source: mBio. 2020 Aug 18;11(4):e01453-20. doi: 10.1128/mBio.01453-20 (PMC7439478; doi:10.1128/mBio.01453-20)

Figure S1

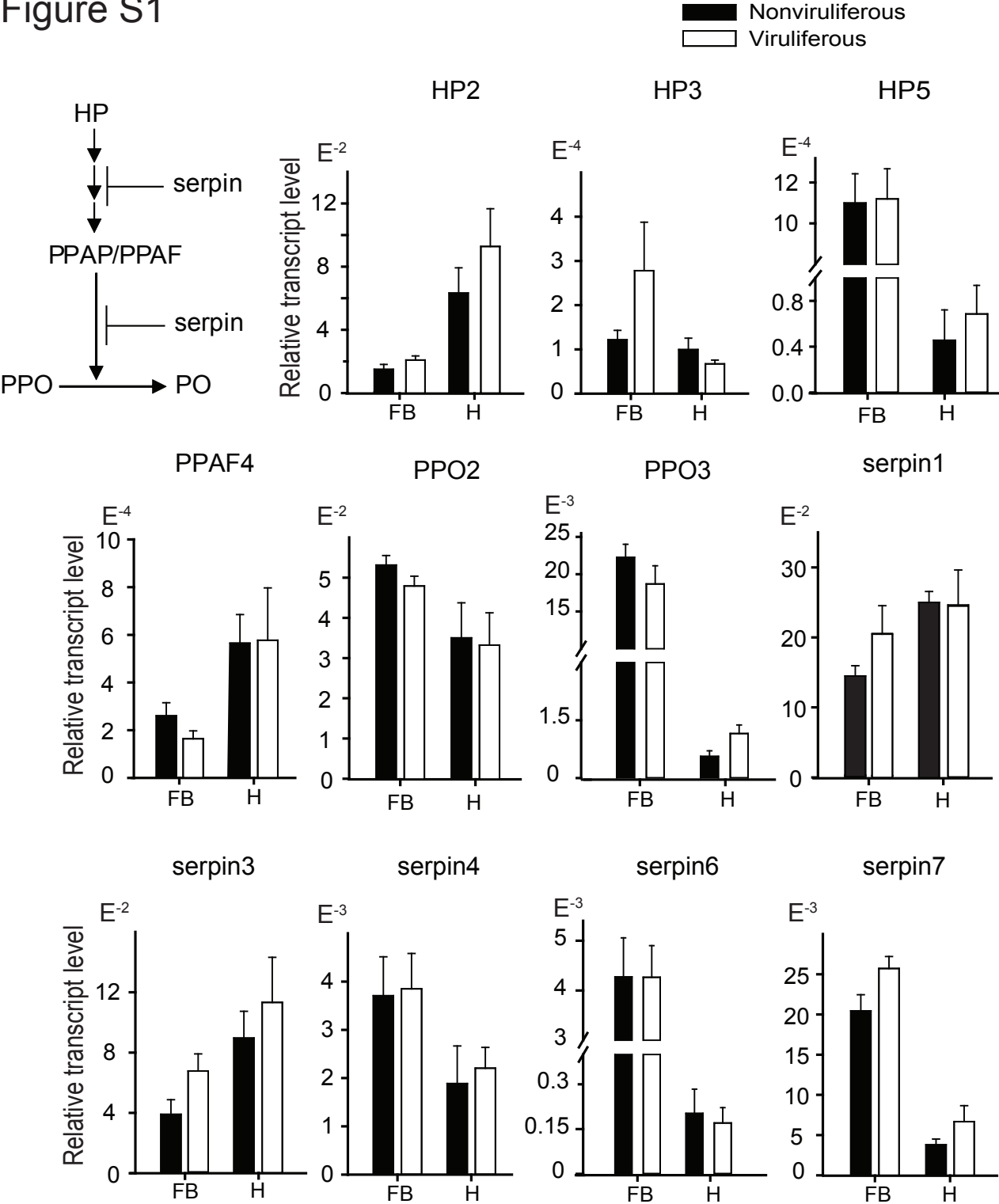

Supplement: FIG S1 [file mBio.01453-20-sf001.pdf]

Figure S2

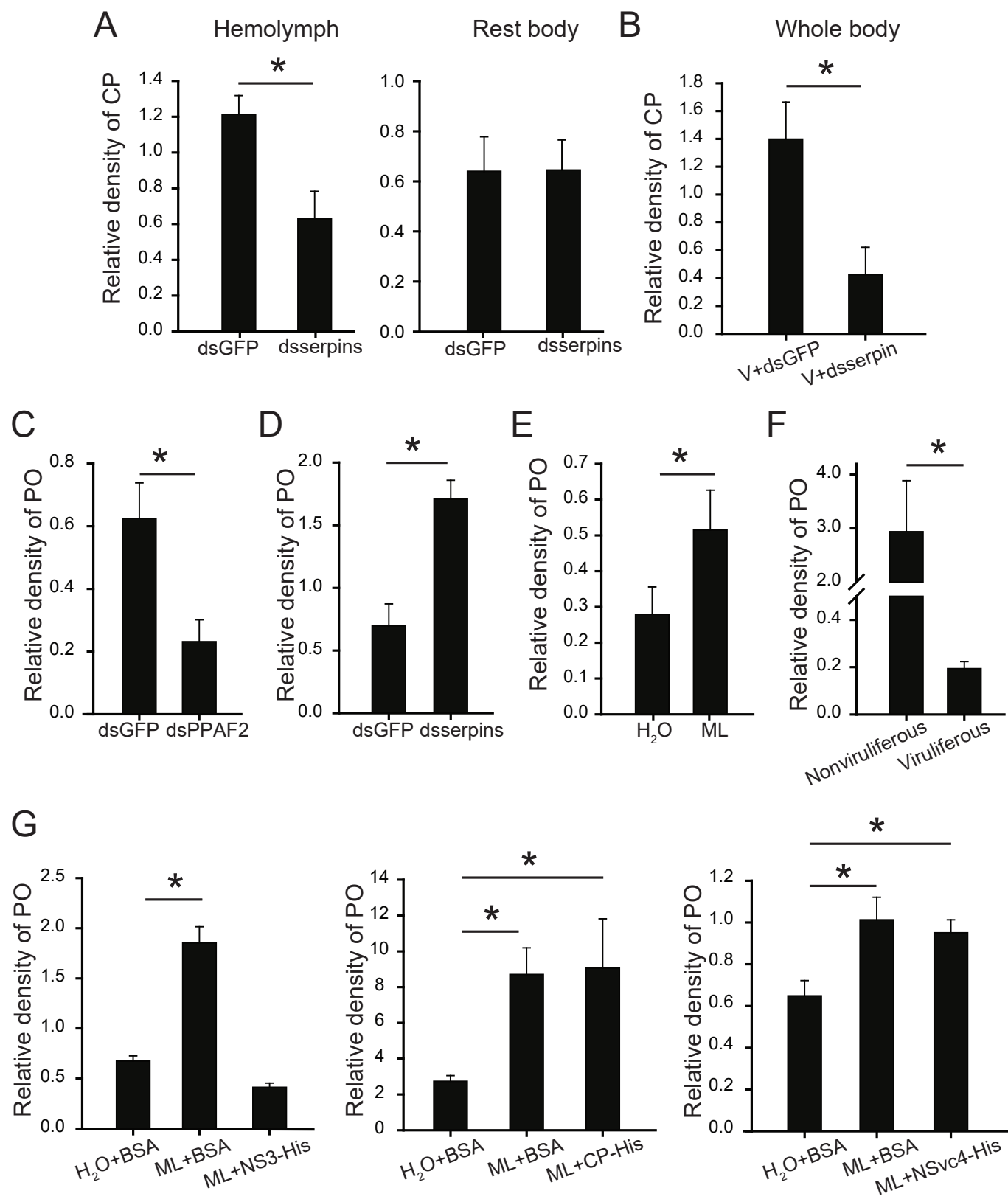

Supplement: FIG S2 [file mBio.01453-20-sf002.pdf]

Figure S3

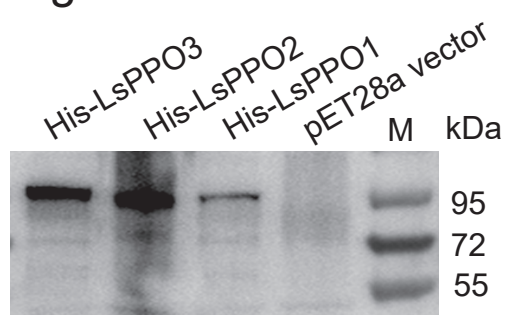

Supplement: FIG S3 [file mBio.01453-20-sf003.pdf]

Figure S4

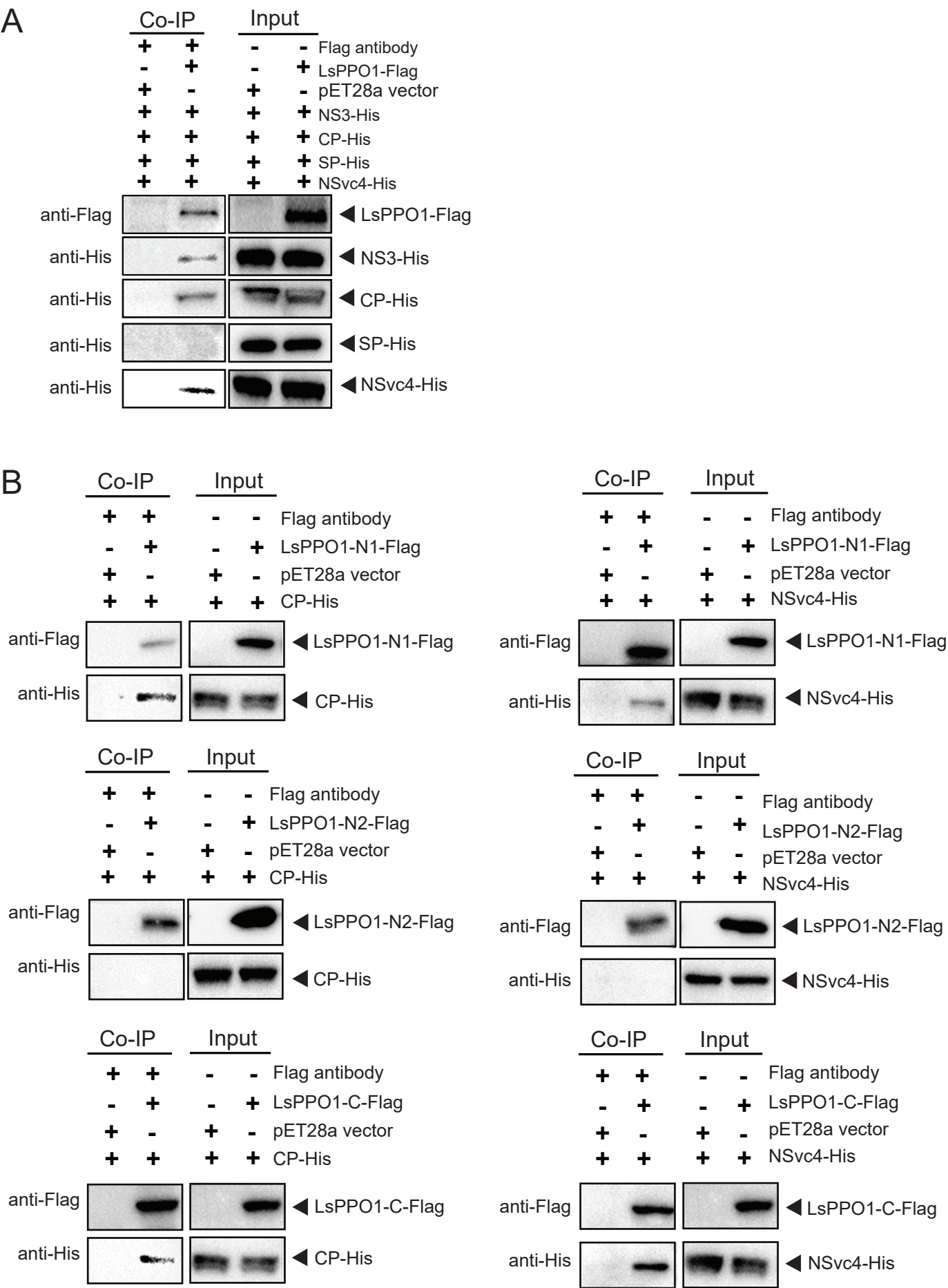

Supplement: FIG S4 [file mBio.01453-20-sf004.pdf]
