# Supplementary material for: A Plant Virus Ensures Viral Stability in the Hemolymph of Vector Insects through Suppressing Prophenoloxidase Activation
Source: mBio. 2020 Aug 18;11(4):e01453-20. doi: 10.1128/mBio.01453-20 (PMC7439478; doi:10.1128/mBio.01453-20)
Supplement: TABLE S1 [file mBio.01453-20-st001.docx]

**Table S1. The GenBank accession numbers of PPO activation cascade members of the small brown planthopper and other insect species**

| Protein name* | Accession number in GenBank |
| --- | --- |
| AaPPAP1 | XP_021711004.1 |
| AaPPAP2 | XP_021711027.1 |
| AaPPAP3 | XP_001650301.1 |
| AaPPO1 | XP_001650638.2 |
| AaPPO2 | XP_001650639.2 |
| AaPPO3 | XP_001661890.1 |
| AaPPO5 | AAV91794.1 |
| Aaserpin1 | XP_001648011.1 |
| Aaserpin2 | XP_021698111.1 |
| AgPPAP2 | ADZ72972.1 |
| AgPPO1 | AAC27383.1 |
| AgPPO4 | CAA09032.1 |
| AgPPO5 | CAA09033.1 |
| AgPPO6 | CAA09034.1 |
| AgPPO7 | CAD31059.1 |
| AgPPO8 | XP_315074.1 |
| AgPPO9 | XP_315076.1 |
| Agserpin2 | XP_308845.4 |
| BmHP21 | NP_001243984.1 |
| BmHP6 | XP_012550963.1 |
| BmPPAF1 | NP_001037053.1 |
| BmPPAP1 | NP_001036832.1 |
| BmPPAP2 | NP_001036844.1 |
| BmPPO1 | NP_001037335.1 |
| BmPPO2 | NP_001037534.1 |
| DmPPAP1 | NP_001303421.1 |
| DmPPAP2 | NP_649734.2 |
| DmPPO1 | NP_476812.1 |
| DmPPO2 | NP_610443.1 |
| DmPPO3 | NP_524760.1 |
| Haserpin5 | ATD13319.1 |
| HcPPO1 | AAC34251.1 |
| HcPPO2 | AAC34256.1 |
| Hcserpin | AAD09285.1 |
| HdHP | Q8I6K0.1 |
| HdPPAF | Q9GRW0.1 |
| HdPPAP | O97366.1 |
| HdPPO1 | Q8I6K1.1 |
| HdPPO2 | Q8I6K2.1 |
| LsHP1 | RZF37131.1 |
| LsHP2 | RZF32993.1 |
| LsHP3 | RZF41609.1 |
| LsHP4 | RZF45073.1 |
| LsHP5 | RZF45072.1 |
| LsHP6 | RZF41612.1 |
| LsHP7 | RZF41610.1 |
| LsPPAF1 | RZF43090.1 |
| LsPPAF2 | RZF34153.1 |
| LsPPAF3 | RZF34672.1 |
| LsPPAF4 | RZF40916.1 |
| LsPPAP1 | RZF43226.1 |
| LsPPAP2 | RZF43227.1 |
| LsPPAP3 | RZF43228.1 |
| LsPPO1 | RZF43154.1 |
| LsPPO2 | RZF35801.1 |
| LsPPO3 | RZF47724.1 |
| Lsserpin1 | RZF49115.1 |
| Lsserpin2 | RZF32069.1 |
| Lsserpin3 | RZF35163.1 |
| Lsserpin4 | RZF34946.1 |
| Lsserpin5 | RZF34947.1 |
| Lsserpin6 | RZF40870.1 |
| Lsserpin7 | MN918594 |
| MsHP2 | AF017664.1 |
| MsHP21 | AAV91019.1 |
| MsHP6 | AAV91004.1 |
| MsHP8 | AAV91006.1 |
| MsPPAF1 | AAM69352.2 |
| MsPPAF2 | AAM69353.1 |
| MsPPAP1 | AAX18636.1 |
| MsPPAP2 | AAL76085.1 |
| MsPPAP3 | AAO74570.1 |
| MsPPO | L42556.1 |
| Msserpin12 | MG732913.1 |
| Msserpin1J | AAC47340.1 |
| Msserpin3 | AAO21505.1 |
| Msserpin4 | AAS68503.1 |
| Msserpin6 | AAV91026.1 |
| Msserpin7 | ADM86478.1 |
| OfPPAP1 | AML39474.1 |
| OfPPO1 | ABC59699.2 |
| OfPPO2a | AQM36768.1 |
| OfPPO2b | AQM36767.1 |
| OfPPO3 | AQM36766.1 |
| Ofserpin3 | AHA43071.1 |
| TmHP | BAG14261.1 |
| TmPPAF1 | BAC15605.1 |
| TmPPAF2 | CAC12696.1 |
| TmPPAP | BAG14262.2 |
| TmPPO | BAA75470.1 |
| Tmserpin40 | BAI59106.1 |
| Tmserpin48 | BAI59108.1 |
| Tmserpin55 | BAI59107.1 |

* Aa, *A. aegypti*; Ag, *A. gambiae*; Bm, *B. mori*; Dm, *D. melanogaster*; Hd, *H. diomphalia*; Ha, *H. armigera*; Hc, *H. cunea*; Ls, *L. striatellus*; Ms, *M. sexta*; Of, *O. furnacalis*; Tm, *T. molitor*.
