# Supplementary material for: A Plant Virus Ensures Viral Stability in the Hemolymph of Vector Insects through Suppressing Prophenoloxidase Activation
Source: mBio. 2020 Aug 18;11(4):e01453-20. doi: 10.1128/mBio.01453-20 (PMC7439478; doi:10.1128/mBio.01453-20)
Supplement: TABLE S2 [file mBio.01453-20-st002.docx]

**Table S2. Primers used in this study**

| Primer name | | Primer sequence | |
| --- | --- | --- | --- |
| HP1-qF | | CGCCAATCTTCTTCTTCAAG | |
| HP1-qR | | GAACCGACTGCTAATCAATG | |
| HP2-qF | | TGCTCAGGTCCTCTTCAA | |
| HP2-qR | | GGTGGAGTCTGGATTCTTC | |
| HP3-qF | | CTACAGATTAGACCGAGAAGT | |
| HP3-qR | | ATCATTGAGTCAGCATCCATG | |
| HP4-qF | | CCTATAACGACATCACACTCT | |
| HP4-qR | | CGACCAGAACTGAGAACAT | |
| HP5-qF | | AGTTGAGCGAAGGAGATTC | |
| HP5-qR | | TCTGTTGTTGCGAAGGTT | |
| HP6-qF | | CGAGTATATTCGTCCTATATGC | |
| HP6-qR | | TATCGTCCGTGAGATTCTTAG | |
| HP7-qF | | TGACGATCCACTAACTTGTT | |
| HP7-qR | | CTCCACCATCTTCTCCATAG | |
| PPAP1-qF | | TTTCAACTTCGACCACC | |
| PPAP1-qR | | CTCGTAATTCGGCTGGT | |
| PPAP2-qF | | ACTGTGAACCGCTCCTAA | |
| PPAP2-qR | | GAGTTATCGTTGTTGCCATT | |
| PPAP3-qF | | CAGCAGCAGTCCAATGAA | |
| PPAP3-qR | | TCTCACAACGGTCAGTCT | |
| PPAF1-qF | | TGAATGCATATGTGTGCC | |
| PPAF1-qR | | TTTCCAACAGCATACGTC | |
| PPAF2-qF | | AAGAAGGCAACTACCAAGT | |
| PPAF2-qR | | GTATTGACCAAGGCGTGTA | |
| PPAF3-qF | | CGACCGCAATATAATTCTCA | |
| PPAF3-qR | | CCTATGTGGATGTTGTAGTTC | |
| PPAF4-qF | | CATTAGGCAGTGAGTGTGA | |
| PPAF4-qR | | GACAAGGAGTTGAGGAAGG | |
| PPO1-qF | | TCGAAGAATCAGGTCAACC | |
| PPO1-qR | | TTGCGTAGACCCATGAGTATG | |
| PPO2-qF | | CTGTCGTCAAAACATGAATC | |
| PPO2-qR | | AGCTGATATTGGCCTCCAT | |
| PPO3-qF | | AACATGAATCCATTGCTGT | |
| PPO3-qR | | ATTAATGTTGGCCTCTTGTC | |
| serpin1-qF | | GAGGAATCACCGATGTAGAA | |
| serpin1-qR | | ACCAACAATAAGACCAGGAT | |
| serpin2-qF | | AGCAGAAGGTCAAGGACT | |
| serpin2-qR | | AATGACCTGCGTATTCTCG | |
| serpin3-qF | | ACTGAAGGAAGGATTAGAAGG | |
| serpin3-qR | | TGAACAGCATCACTCACAA | |
| serpin4-qF | | CGAACGGTAGAATCAAGGT | |
| serpin4-qR | | GAGGAACACTATCGCTATCA | |
| serpin5-qF | | GAGACAATACAACATCCTACG | |
| serpin5-qR | | CAAGCTCGGCATCTGATT | |
| serpin6-qF | | ACACTGTTCTCGCCAATC | |
| serpin6-qR | | ACTGTTACTTCTGAGCCATT | |
| serpin7-qF | | TCGTTTGTGGATGTGCTG | |
| serpin7-qR | | ACCGTTGAAGAAGATCAC | |
| CP-qF | | GATGAAGTACACAACTGGTC | |
| CP-qR | | AGTGCTGATCGTATTGACAGA | |
| EF2-qF | | GTCTCCACGGATGGGCTTT | |
| EF2-qR | | ATCTTGAATTTCTCGGCATACATTT | |
| dsGFP-F | | CACAAGTTCAGCGTGTCCG | |
| dsGFP-R | | GTTCACCTTGATGCCGTTC | |
| dsGFP-T7F | | TAATACGACTCACTATAGGCACAAGTTCAGCGTGTCCG | |
| dsGFP-T7R | | TAATACGACTCACTATAGGGTTCACCTTGATGCCGTTC | |
| dsPPAF2-F | | AGAAGGACAACGAACACTT | |
| dsPPAF2-R | | GAAGGCGTTCTCCAGATAG | |
| dsPPAF2-T7F | | TAATACGACTCACTATAGGAGAAGGACAACGAACACTT | |
| dsPPAF2-T7R | | TAATACGACTCACTATAGGGAAGGCGTTCTCCAGATAG | |
| dsserpin2-F | | TGTTGCAAACTGTAAACCAC | |
| dsserpin2-R | | TTCCGCTGACTTGGCAAAG | |
| dsserpin2-T7F | | TAATACGACTCACTATAGGTGTTGCAAACTGTAAACCAC | |
| dsserpin2-T7R | | TAATACGACTCACTATAGGTTCCGCTGACTTGGCAAAG | |
| dsserpin7-F | | CAAGTTCGATATATTCGACTGG | |
| dsserpin7-R | | TTGCTTTCGATACCTCGCTG | |
| dsserpin7-T7F | | TAATACGACTCACTATAGGCAAGTTCGATATATTCGACTGG | |
| dsserpin7-T7R | | TAATACGACTCACTATAGGTTGCTTTCGATACCTCGCTG | |
| dsCP-F | | AACAAGCCAGCCACTCTA | |
| dsCP-R | | TCCACAGCCATCTTAACAC | |
| dsCP-T7F | | TAATACGACTCACTATAGGAACAAGCCAGCCACTCTA | |
| dsCP-T7R | | TAATACGACTCACTATAGGTCCACAGCCATCTTAACAC | |
| T-NS3-F | | ATGAACGTGTTCACATCG | |
| T-NS3-R | | CTACAGCACAGCTGGAG | |
| T-CP-F | | ATGGGTACCAACAAGC | |
| T-CP-R | | CTAGTCATCTGCACCTTC | |
| T-SP-F | | ATGCAAGACGTACAAAGG | |
| T-SP-R | | CTATGTTTTATGAAGAAG | |
| T-NSvc4-F | | ATGGCTTTGTCTCGACTT | |
| T-NSvc4-R | | CTACATGATGACAGA | |
| T-LsPPO1-F | | ATGGCCAACAAGAACCGA | |
| T-LsPPO1-R | | GTTTCTCTTGGGGG | |
| T-LsPPO2-F | | ATGGCTTTGAATGAAATAT | |
| T-LsPPO2-R | | TTAGGTTCTAGAAACCG | |
| T-LsPPO3-F | | ATGGCTGACACCACCAA | |
| T-LsPPO3-R | | TTAGTTGCGTGGCCTGTT | |
| T-lipoprotein-antigen(39-600)-F | | TATGAAGAAGGCGCTAGCTAC | |
| T-lipoprotein-antigen(39-600)-R | | TTAGAACTTGTTCTTGGTGTAGATG | |
| pET28a-NS3-F | | ATGGGTCGCGGATCCATGAACGTGTTCACATCG | |
| pET28a-NS3-R | | GGTGGTGGTGCTCGAGCTACAGCACAGCTGGAG | |
| pET28a-CP-F | | ATGGGTCGCGGATCCATGGGTACCAACAAGC | |
| pET28a-CP-R | | GGTGGTGGTGCTCGAGCTAGTCATCTGCACCTTC | |
| pET28a-SP-F | | ATGGGTCGCGGATCCATGCAAGACGTACAAAGG | |
| pET28a-SP-R | | GGTGGTGGTGCTCGAGCTATGTTTTATGAAGAAG | |
| pET28a-NSvc4-F | | ATGGGTCGCGGATCCATGGCTTTGTCTCGACTT | |
| pET28a-NSvc4-R | | GGTGGTGGTGCTCGAGCTACATGATGACAGA | |
| pET28a-His-LsPPO1-F | | ATGGGTCGCGGATCCATGGCCAACAAGAACCGA | |
| pET28a-His-LsPPO1-R | | GGTGGTGGTGCTCGAGTTAGTTTCTCTTGGGGG | |
| pET28a-His-LsPPO2-F | | ATGGGTCGCGGATCCATGGCTTTGAATGAAATAT | |
| pET28a-His-LsPPO2-R | | GGTGGTGGTGCTCGAGTCAGGTTCTAGAAACCG | |
| pET28a-His-LsPPO3-F | | ATGGGTCGCGGATCCATGGCTGACACCACCAA | |
| pET28a-His-LsPPO3-R | | GGTGGTGGTGCTCGAGCTAGTTGCGTGGCCTGTT | |
| pET28a-His-lipoprotein-antigen(39-600)-F | | ATGGGTCGCGGATCCTATGAAGAAGGCGCTAGCTAC | |
| pET28a-His-lipoprotein-antigen(39-600)-R | | GGTGGTGGTGCTCGAGTTAGAACTTGTTCTTGGTGTAGATG | |
| pET28a-His-LsPPO1-antigen(174-500)-F | | ATGGGTCGCGGATCCCCAATTGAGATCCCGAT | |
| pET28a-His-LsPPO1-antigen(174-500)-R | | GGTGGTGGTGCTCGAGTTAGCGAACGGTGCCCTGTC | |
| pET28a-Flag-LsPPO1-F | | GGAGATATACCATGGGCATGGCCAACAAGAACCGA | |
| pET28a-Flag-LsPPO1-R | | ACGGAGCTCGAATTCTTACTTGTCATCGTCGTCCTTGTAGTCGTTTCTCTTGGGGG | |
| pET28a-Flag-LsPPO1-N1-R | | ACGGAGCTCGAATTCTTACTTGTCATCGTCGTCCTTGTAGTCCCAGTGCCAGTGGTGCAA | |
| pET28a-Flag-LsPPO1-N2-F | | GGAGATATACCATGGGCAACCTAGACGACCTGGAG | |
| pET28a-Flag-LsPPO1-C-F | | GGAGATATACCATGGGCCATCTCGTCTATCCGTTC | |
| pET28a-Flag-LsPPO3-N2-F | | GGAGATATACCATGGGCGGCATTGAGGATTTCATA | |
| pET28a-Flag-LsPPO3-N2-R | | ACGGAGCTCGAATTCTTACTTGTCATCGTCGTCCTTGTAGTCCCAATGCCAGTGATGCAA | |
